# Respectful maternity care and associated factors among mothers who gave birth in three hospitals of Southwest Ethiopia: A cross-sectional study

**DOI:** 10.3389/fpubh.2022.1055898

**Published:** 2023-01-04

**Authors:** Amanuel Adugna, Kassa Kindie, Gossa Fetene Abebe

**Affiliations:** ^1^Department of Midwifery, Mizan Tepi University, Mizan Teferi, Ethiopia; ^2^Department of Nursing, Mizan Tepi University, Mizan Teferi, Ethiopia

**Keywords:** abuse, disrespect, Mizan Tepi University, respectful maternity care, associated factors

## Abstract

**Background:**

One of the primary barriers to reducing maternal morbidity and mortality is disrespect and abuse during childbirth in biomedical facilities. Despite the serious consequences of disrespect and abuse during childbirth, there is no evidence of the prevalence of respectful maternity care in Southwest Ethiopia. This study aimed to assess the prevalence of respectful maternity care and associated factors among mothers who gave birth in three hospitals in Southwest Ethiopia.

**Methods:**

An institution-based cross-sectional study was conducted among 348 mothers who gave birth in three hospitals in Southwest Ethiopia. Bivariable and multivariable binary logistic regression were used to identify the factors of respectful maternity care.

**Results:**

In this study, 348 mothers with their newborns were included, making a response rate of 100%. The overall prevalence of respectful maternity care was 81.2%. Maternal age [AOR = 2.54; 95% CI (1.01–6.43)]; maternal occupation [AOR = 5.23; 95% CI (1.15–23.72)]; antenatal care follows-up [AOR = 2.86; 95% CI (1.01–8.20)]; and discussions with the provider about the place of delivery during antenatal care follow up [AOR = 5.58; 95% CI: (2.12–14.70)] were found to be the most significant components of respectful maternity care.

**Conclusion:**

The provision of respectful maternity care was high, but there are complaints of disrespect and abuse still present in three hospitals in Southwest Ethiopia. Maternal age, maternal occupation, antenatal follow up, and discussion with the provider about the place of delivery during antenatal follow-up were associated with respectful maternity care. Thus, improving antenatal care service utilization and discussions with health care providers about the place of delivery during antenatal care follow-up should be focused on.

## Introduction

Pregnancy, labor, and delivery are crucial events in the lives of women and their families in all countries (1). The disrespect and abuse perpetrated on women in biomedical health facilities during childbirth is a global problem (2, 3). Respectful maternity care (RMC) is a person-centered strategy for maternity service that encourages procedures that reflect women's preferences and newborns' needs. It is based on ethical principles and respect for human rights (4, 5). We define disrespect and abuse (D&A) as any harsh care or thoughtless conduct directed toward a woman during childbirth (6).

Disrespectful maternity care has both direct and indirect impacts on women's birth outcomes, patient satisfaction, and future delivery plans (3, 7, 8).

The Ethiopian Minister of Health has launched a new compassionate, respectful maternity care (CRC) program based on WHO principles during childbirth and has given in-service training to all maternity care providers on CRC (9, 10). In addition, the establishment of maternal waiting rooms in biomedical health facilities, free labor and delivery service and transportation, pregnant women's conferences, and a media awareness campaign have all been attempted to promote SBA delivery (11). Despite these actions, 72% of Ethiopian women deliver their children at home, with a range of indicators depending on geography, economic status, and education levels (12). In addition, maternal and neonatal mortality rates remain at 412 per 100,000 and 29 per 1,000 live births, respectively (13). Furthermore, disrespectful maternity care at biomedical healthcare facilities is one of the key reasons for home delivery (14).

Despite RMC being the best and the key strategy to increase institutional delivery and reduce maternal and newborn mortality, there is no evidence of the prevalence and risk factors of RMC in the study area. Thus, this study aimed to assess the RMC and associated factors among women who gave birth in three public hospitals in Southwest Ethiopia.

## Methods and materials

### Study area

The study was conducted at Mizan-Tepi University Teaching Hospital (MTUTH), Gebretsadik-Shawe General Hospital (GSGH), and Tepi General Hospital (TGH) in Southwest Ethiopia. Those hospitals are the only hospitals in the Southwest Ethiopia regional state and are located in Benchi-Sheko Zone, Kefa Zone, and Sheka Zone, respectively. The hospitals provide emergency obstetric care, maternal and neonatal health infrastructures, and a full complement of skilled health professionals, including obstetricians, anesthesiologists, radiologists, neonatologists, nurses, and midwives.

### Study design, and period

A facility-based cross-sectional study was conducted in these hospitals from 1 January to 30 February 2021.

### Population

The study population included women who gave birth in three hospitals in Southwest Ethiopia, while the source population included mothers who gave birth in three hospitals in Southwest Ethiopia during the study period.

### Eligibility criteria

Mothers in the postnatal care room during the study period were included in the study, whereas mothers who were critically ill and unable to communicate were excluded.

### Sample size determination and sampling procedure

The sample size was calculated using the single population proportion formula based on the assumptions below. The 95% confidence interval (CI), (Z/2 = 1.96), marginal error (d) = 0.05, and proportion of RMC *p* = 71% are based on a study conducted in Jimma, Ethiopia. As a result, 348 mothers were included in the study after allowing for a 10% non-response rate (15).

The study was conducted simultaneously at MTUTH, GSGH, and TGH. A proportional sample allocation was applied to each hospital based on the number of deliveries performed in the 2 months before data collection. A total of 348 mothers (139, 113, and 96 from MTUTH, GSGH, and TPGH, respectively) participated in the study. For each hospital, the study participants were identified by systematic random sampling technique from the postnatal registration book. Participants were recruited within 12 h of delivery in the postnatal room.

### Operational definitions

#### Skilled birth attendant

An accredited health professional such as a midwife, doctor, or nurse who has been educated and trained to proficiency in the skills needed to manage normal pregnancy, childbirth, and the immediate postnatal period, and in the identification, management, and referral of complications in women and newborns (16).

### Measurements

The mothers were interviewed and their medical records were reviewed using an Amharic version interviewer-administered questionnaire and an English version checklist. Previous literature was used to construct the structured questionnaire and extraction checklist (17).

For the prevalence of RMC, 15 interviewer-administered questionnaires that contain dichotomous (yes/no) questions were used. It was determined that a mother was receiving RMC if she answered yes to all 15 questions. If a mother responded negatively (no) to at least one question, we have considered that she had disrespectful delivery care (17).

The data on maternal age, religion, marital status, occupation, educational level, residence, income, ethnicity, antenatal care (ANC) follow-up, the reason to deliver in the hospital, admission to the maternity waiting room before labor started, the duration of labor, the gender of the maternity care provider attending the birth, and a face-to-face interviewer-administered questionnaire in the postnatal room were used to collect information from providers on the place of birth during ANC. Healthcare providers attending labor and delivery, companion during labor, labor outcome, and labor started spontaneously/ induced were collected an extract checklist used when reviewing their medical records.

### Data quality control

Technical training for data collectors was provided before the actual data collection, and a pretest on 5% of the total sample size outside the study area with similar characteristics to the study population was conducted to ensure the quality of the data. The adapted English questionnaire was translated into Amharic, the local language, and then back to English by a language expert for consistency checks. We used six trained and degreed midwives as data collectors and two physicians as supervisors to collect all the requisite sample sizes. Each completed questionnaire was double-checked for accuracy, consistency, and completeness by the supervisors every day.

### Data processing and analysis

The data were double-checked, coded, and entered into Epi Data version 3.1 software packages before being exported to SPSS software for analysis. Frequency tables and percentages were also used to present the descriptive analysis. To explain the data, the mean and standard deviations were calculated and used for applicable variables. For the multivariable regression model, bivariable regression analysis with a *p* < 0.25 was used to select candidate variables. The AOR with their 95% confidence CI was calculated after a multivariable logistic regression was fitted using the centering method. Finally, variables in the multivariable regression analysis with a *p* < 0.05 were considered significant.

## Results

### Sociodemographic characteristics

A total of 348 mothers with newborns were included in the study, making a response rate of 100%. 149 (42.8%) of respondents fall in the 20–24 years age group. Nearly one-third of the study participants, 133 (38.2%) were from the Bench ethnic group and 149 (42.8%) were followers of the Protestant religion. About two-thirds of the mothers, 257 (73.9%), were married. Around half of them, 201 (57.8%) were housewives, and one-third of them, 112 (32.2%), had a monthly family income of 10–30 US dollars (USD). Above half of the respondents, 200 (57.5%) were non-educated (Table 1).

**Table 1:** The socioeconomic characteristics of mothers at three public hospitals in Southwest Ethiopia in 2021 (*n* = 348).

**Variables**	**Categories**	**Number of respondents**	**Percentage**
Age	20	66	19
	20–34	149	42.8
	> 35	133	38.2
Marital status	Single	27	7.7
	Married	257	73.8
	Divorced	25	7.2
	Widowed	39	11.3
Religion	Ethiopian orthodox	128	36.8
	Protestant	149	42.8
	Muslim	71	20.4
Ethnicity	Bench	133	38.2
	Shako	36	10.3
	Kaffa	82	23.6
	Oromo	37	10.6
	Amhara	60	17.3
Occupation	Housewife	201	57.7
	Merchant	70	20.1
	Private employee	34	9.8
	Government employee	43	12.4
Educational status	Non-educated	200	57.5
	Primary	100	28.7
	Secondary	36	10.4
	College and above	12	3.4
Family monthly income	<10 USD	96	27.6
	10–30 USD	112	32.2
	30–40 USD	76	21.8
	≥40 USD	64	18.4

### Obstetric related characteristics

Of the total respondents, 317 (91.1%) had a history of ANC follow-up for recent childbirth; 266 (76.4%) of the mothers were attended by a midwife; 271 (77.9%) of mothers gave birth through spontaneous vaginal delivery, and more than half of 201 (57.8%) healthcare providers were males. The majority of mothers (281) (80.7%) were admitted to maternity waiting homes, which are designed to improve access to obstetric care for high-risk pregnant women or those who live very far away from health facilities before delivery. More than half, 184 (52.9%), mothers discussed delivery with a provider, and 276 (79.3%) did not have complications during delivery (Table 2).

**Table 2:** The obstetric characteristics of mothers in three public hospitals in Southwest Ethiopia in 2021 (*n* = 348).

**Variables**	**Categories**	**Number of respondents**	**Percentage**
ANC follow up	Yes	317	91.1
	No	31	8.9
Healthcare providers conducting labor and delivery	Nurse	25	7.2
	Midwife	266	76.4
	Doctor	57	16.4
Sex of health care provider conducting labor and delivery	Male	201	57.8
	Female	147	42.2
Companion during labor	Yes	72	20.7
	No	276	79.3
Labor outcome	Normal	293	84.2
	With complication	55	15.8
Reason to deliver in the hospital	Planned	182	52.3
	Referral	166	47.7
Admitted to maternity waiting home before labor started	Yes	281	80.7
	No	67	19.3
Duration of labor	<12 h	210	60.3
	>12 h	138	39.7
Labor started	Spontaneously	212	60.9
	Induced	136	39.1
Discussion with providers about the place of delivery	Yes	184	52.9
	No	164	47.1

### Proportion of RMC

**Figure 1:**
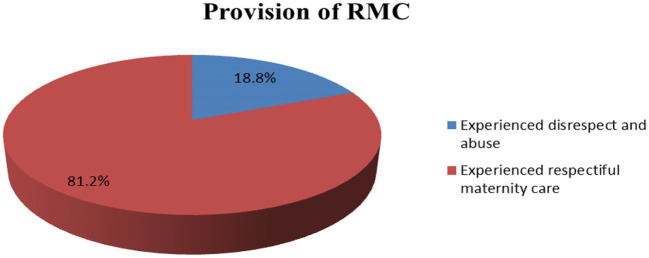
Provision of RMC during labor and delivery in three public hospitals of Southwest Ethiopia (*n* = 348).

Of the proportion of RMC out of 348 mothers who gave birth in three public hospitals in Southwest Ethiopia, 283 (81.2%) mothers received RMC, but the remaining 65 (18.8) mothers were disrespected during labor and delivery (Figure 1). Out of the 348 respondents, 82.6%, 77.0%, 84.1% and 79.3% received friendly, abuse-free, timely, and discrimination-free care, respectively (Table 3).

**Table 3:** Provision of RMC during childbirth at three public hospitals in Southwest Ethiopia, 2021 (*n* = 348).

**Category of RMC**	**Items of RMC**	**Experienced RMC**			
		**Yes**	**Percentage (%)**	**No**	**Percentage (%)**
Friendly care	Care with a kind approach	299	85.9	49	14.1
	Care in a friendly manner	289	83.0	59	17.0
	Care with positive talk about pain and relief	293	84.2	55	15.8
	Care with showing empathy	286	82.2	62	17.8
	Care with respect to individual	302	86.8	46	13.2
	Care with understandable language	278	79.9	70	20.1
	Care by calling the laboring woman by the name	265	76.1	83	23.9
	Overall average	82.6%		17.4%	
Abuse-free care	The HWs responded to my needs, whether or not I asked.	254	73.0	94	27.0
	No one slapped me during delivery for different reasons.	293	84.2	55	15.8
	No one shouted at me because I haven't done what I was told to do.	257	73.9	91	26.1
	Overall average	77.0%		23.0%	
Timely Care	I didn't have to wait long before receiving service.	297	85.3	51	14.7
	Service provision was not delayed because of the health facility's internal problem	289	83.0	59	17.0
	Overall average	84.1%		15.9%	
Discrimination- free care	No one treats me disrespectfully due to some personal attributes	281	80.8	67	19.2
	No one insulated me due to personal attributes	289	83.0.0	59	16.1
	No one discriminated against me by economic status and language	285	81.9	63	18.1
	No one discriminated against me by race and ethnicity	276	79.3	72	20.7
	Overall average	81.2%		18.8%	

### Bivariable and multivariable logistic regression analysis

In the bivariable logistic regression model, maternal age, maternal occupation, ANC follow-up, the provider who attended the labor and delivery, companion during labor, labor outcome, a reason to deliver in the hospital, admission to maternity waiting home before labor started, duration of labor, how labor started (spontaneously vs. induced) and discussion about the place of delivery with a provider during ANC follow up were the main components of RMC. After adjusting for confounding variables in a multivariable logistic regression model, maternal age, maternal occupation, ANC follow up and discussion about the place of delivery with a provider during ANC follow-up were identified as the significant predicates of RMC.

Mothers who were <20 years old were 2.5 times more likely to receive respectful maternity care than those whose age was more than or equal to 35 years [AOR = 2.54; 95% CI (1.01–6.43)]. Mothers who had private work were 5.2 times more likely to receive respectful maternity care than women who had governmental work [AOR = 5.23; 95% CI (1.15–23.72)]. Mothers who had at least one history of ANC follow-up in their current pregnancy were 2.8 times more likely to receive RMC as compared to their counterparts [AOR = 2.86; 95%CI (1.01–8.20)]. The odds of experiencing RMC were higher among mothers who discussed the place of delivery with healthcare providers during their current pregnancy as compared to their counterparts (AOR = 5.58; 95% CI: [2.12–14.70]) (Table 4).

**Table 4:** Bivariable and multivariable analyses showing crude and adjusted odds ratios for an association between RMC and risk factors among mothers who give birth in three public hospitals in Southwest Ethiopia, 2021 (*n* = 348).

**Variable**	**Category**	**RMC**		**COR (95% CI)**	**AOR (95% CI)**

		**Yes**	**No**		
Maternal age	<20	46	20	2.13 (1.14–4.67)	2.54 (1.01–6.43)^*^
	20–34	127	22	0.92 (0.48–1.17)	1.03 (0.46–2.33)
	≥35	112	21	1	
Maternal occupation	Housewife	165	36	1.65 (0.61–4.50)	1.62 (0.47–5.56)
	Merchant	59	11	1.41 (0.45–4.40)	1.43 (0.35–5.71)
	Private	23	11	3.63 (1.12–11.79)	5.23 (1.15–23.72)^*^
	Government employee	38	5	1	
ANC follow up	Yes	268	49	4.50 (2.08–9.72)	2.86 (1.00–8.20)^*^
	No	17	14	1	
A provider who conducts delivery	Nurse	39	27	8.07 (2.25–28.96)	2.74 (0.54–13.89)
	Midwife	211	33	1.82 (0.53–6.27)	1.13 (0.27–4.74)
	Doctor	35	3	1	
Companion during labor	Yes	42	30	5.26 (2.90–9.51)	1.43 (0.38–5.36)
	No	243	33	1	
Labor outcome	Normal	254	39	5.04 (2.68–9.47)	0.36 (0.68–1.90)
	With complication	31	24	1	
Reason to deliver in the hospital	Planned	164	18	3.36 (1.86–6.14)	0.63 (0.24–1.65)
	Referral	121	45	1	
Admitted to maternity waiting home before labor started	Yes	224	57	2.58 (1.06–6.28)	2.65 (0.81–8.62)
	No	61	6	1	
Duration spent on labor	<12 h	158	52	3.80 (1.90–7.58)	1.40 (0.38–5.13)
	> 12 h	127	11	1	
Labor started	Spontaneously	158	56	6.61 (2.91–15.01)	2.27 (0.62–8.26)
	Induced	129	7	1	
Discussion with providers about the place of delivery during ANC follow up	Yes	162	22	2.45 (1.39–4.33)	5.58 (2.12–14.70)^*^
	No	123	41	1	

1-Reference, ^*^-Significant at <0.05. COR, Crude odd ratio; AOR, Adjusted odd ratio.

## Discussion

An important approach to minimizing maternal and neonatal morbidity and mortality is to increase pregnant women's access to skilled care during childbirth (5). The study aimed to assess the provision of RMC and related characteristics among birthgiving mothers in three public hospitals in Southwest Ethiopia.

The overall provision of RMC was 81.2%, according to the findings of this study. This result is higher than the studies found in Ethiopia (57%), and Kenya (80 %) (18, 19). However, the result is lower than a study conducted in Tanzania (20). This difference could be due to time differences associated with current accelerated reproductive health promotion activities, women-friendly programs, and various supportive types of training in some health institutions in the study area, as well as the small sample size of previous studies and different study areas.

In the current study, a variety of factors affected RMC. In this regard, the study found that mothers who were younger than 20 years old were more likely to have RMC than older mothers. This finding was supported by research conducted in Kenya (19). The reason for this association could be that women under the age of 20 are more educated than their counterparts; these mothers may have a greater understanding of healthcare facilities, as well as of their rights and privileges.

Mothers who had at least one history of ANC follow-up in their current pregnancies were more likely to receive RMC as compared to their counterparts. This finding is in line with findings from recent studies in Ethiopia's southern and eastern regions (21, 22). ANC follow-up enhances RMC provision. The pregnant women's adaptation to the services and close relationships with the healthcare staff are critical in creating trust in the facility's services.

Last but not least, RMC was part of a pregnant woman's discussion with healthcare providers about the place of birth. In this context, women who discussed a birthplace with maternity care providers during ANC were more likely to receive RMC than those who did not. This evidence supports research conducted in Oromia, Ethiopia, which found that mothers who had not received ANC visits were more likely to be disrespected and abused than those who had received ANC visits (23). This could be because women who had ANC and discussed a birthplace were more likely to know the healthcare professionals who would attend them during their labors and births.

The provision of RMC was high, but there are complaints of disrespect and abuse still present in three hospitals in Southwest Ethiopia. Maternal age, maternal occupation, antenatal follow up and discussion with the provider about the place of delivery during antenatal follow-up were associated with respectful maternity care. Thus, improving ANC service utilization and discussion with healthcare providers about the place of delivery during ANC follow-up should be focused on.

### Study limitations

Our research might have the following limitations: Our research might have been subject to social desirability bias and fear of reporting abusive care, since the data were collected within the hospital setting. The other limitation is that meanwhile, the data were collected in the early postpartum period; thereby some women were too exhausted to respond to some questions.

## Data availability statement

The raw data supporting the conclusions of this article will be made available by the authors, without undue reservation.

## Ethics statement

The studies involving human participants were reviewed and approved by Mizan Tepi University. The patients/participants provided their written informed consent to participate in this study.

## Author contributions

AA developed the conception of the idea, wrote the proposal, and participated in the data collection and analysis. KK participated in data analysis, report writing, and prepared the manuscript. GA approved the proposal with some revisions and participated in manuscript development. All authors read and approved the final manuscript.
